# The effect of single dose of gonadotropin-releasing hormone agonist injection in frozen-thawed embryo transfer on pregnancy outcomes: A systematic review and meta-analysis

**DOI:** 10.5935/1518-0557.20240054

**Published:** 2024

**Authors:** Pongpawan Chienvichai, Natpat Jansaka, Usanee Sanmee, Kittipat Charoenkwan

**Affiliations:** 1 Department of Obstetrics and Gynecology, Faculty of Medicine, Chiang Mai University, Chiang Mai, Thailand

**Keywords:** gonadotropin-releasing hormone agonist, luteal phase support, frozen-thawed embryo transfer, pregnancy rate

## Abstract

This systematic review and meta-analysis of randomized controlled trials aimed to evaluate the effect of a single-dose gonadotropin-releasing hormone agonist administration in the frozen-thawed embryo transfer cycle on pregnancy outcomes. A literature search was strategically conducted using PubMed, EMBASE, and the Cochrane Controlled Trials Register. The primary outcome was the clinical pregnancy rate. The secondary outcomes combined chemical pregnancy rate, implantation rate, ongoing pregnancy rate, live birth rate, miscarriage rate, and extrauterine pregnancy rate. Out of the 1594 citations that were found, only six met the criteria for being included in the meta-analysis. The clinical pregnancy rate was higher in the treatment group than in the control group (52.05% *vs.* 47.29%; *p*=0.04; RR=1.09; 95% CI=1.00-1.18). According to subgroup analysis based on the natural cycle, the clinical pregnancy rate with the agonist administration is significantly higher (43.75% vs. 27.35%; *p*=0.01; RR=1.6; 95% CI=1.10-2.32). However, there was no difference between the groups in terms of artificial cycles (*p*=0.80; 95% CI=0.96-1.20). The secondary outcomes did not show significant differences. We concluded that supplementing with a single dose of gonadotrophin-releasing hormone agonist can marginally increase the clinical pregnancy rate, particularly in the natural cycle. Other pregnancy outcomes do not improve with the treatment.

## INTRODUCTION

Freeze-all protocol along with frozen-thawed embryo transfer (FET) strategies is expanding substantially because of effective cryopreservation techniques and convenience. This technique reduces the risk of ovarian hyperstimulation syndrome and increases endometrial receptivity for embryos. Moreover, the duration of the preimplantation genetic testing process is allowed before the transfer (Singh *et al.,* 2020). Several studies revealed that live birth and clinical pregnancy rates were significantly increased in the FET compared with fresh cycle transfer (Shapiro *et al.,* 2011; 2013; 2014; Özgür *et al.,* 2015). Consequently, there is a tendency toward using embryo freezing with FET rather than fresh transfer. One metaanalysis suggests that FET was shown to lower the risk of poor neonatal outcomes such as preterm birth and low birth weight (Sha *et al.,* 2018).

FET should be done during an appropriate endometrial window, because synchronization between the developing embryo and receptive endometrium is required to achieve implantation (Fazleabas & Strakova, 2002). Before the FET, endometrial preparation methods which consist of natural cycle, modified natural cycle, and artificial cycle were opted and applied. It is still undetermined which endometrial preparation protocol is more beneficial to yield a high live birth rate (Groenewoud *et al.,* 2013). However, the artificial cycle is scheduled and less monitored.

Currently, evidence has suggested gonadotrophin-releasing hormone (GnRH) activity in the conceptus. It was reported that identified GnRH receptors in embryos at the morula and blastocyst stages reflect a role for GnRH in embryonic development and probable implantation (Casañ *et al.,* 1999; Kawamura *et al.,* 2005). Additionally, GnRH and its receptor expression have been detected in endometrium, with greater expression in epithelial cells during the luteal phase than in the proliferative phase (Raga *et al.,* 1998).

Several studies have discovered that a single dose of GnRH agonist (GnRHa) during the luteal phase has a positive influence on pregnancy outcomes such as implantation, clinical pregnancy, and live birth (Tesarik *et al.,* 2004; 2006; Isik *et al.,* 2009; Razieh *et al.,* 2009). Recent meta-analyses have shown some inconsistent results regarding the benefit of GnRHa as a result of different embryo transfer protocols and various regimens of GnRH administrations (Oliveira *et al.,* 2010; Martins *et al.,* 2016; Li & Li, 2018; Chau *et al.,* 2019; Song *et al.,* 2020). Furthermore, no prior meta-analyses have intended to evaluate the effect of GnRHa in the FET cycle and emphasize randomized controlled trials (RCTs) with single-dose regimens.

The purpose of this systematic review and metaanalysis was to summarize the data from RCTs to assess the impact of GnRHa given once for the FET cycle on pregnancy outcomes.

## MATERIALS AND METHODS

### Protocol Registration

This systematic review protocol was registered at the International Prospective Register of Systematic Reviews (PROSPERO) and accepted with registration number CRD42021291651. Since this is a systematic review, this protocol is exempt from review by the Research Ethics Committee of the Faculty of Medicine, Chiang Mai University.

### Eligibility Criteria

All published or abstract reports of RCTs, including parallel group and cross-over studies, were considered eligible for review. When cross-over trials were included, all data from all treatment protocols for each participant were analyzed. All RCTs that assess pregnancy outcomes after receiving an additional single dose of GnRHa injection compared with practical luteal phase support during FET cycles regardless of endometrial preparation technique. Any RCTs including fresh embryos or more than one dose of GnRHa injection were excluded.

### Outcome Measures

The primary pregnancy outcome was clinical pregnancy rate. Secondary outcomes consisted of positive pregnancy rates (or other similar terms used in the studies), miscarriage rates, implantation rates, ongoing pregnancy rates, live birth rates, and extrauterine pregnancy rates (or ectopic pregnancy).

### Search Strategy and Study Selection

For the search strategy, an online literature search of databases in EMBASE, PubMed, and Cochrane Controlled Trials Register (CENTRAL) was conducted, with no language limitation, from the date of database inception to March 31, 2022. These terms were used: ((frozen-thawed) OR (frozen) OR (freezing) OR (freeze) OR (cryopreservation) OR (cryopreservative)) AND (embryo transfer) AND ((GnRH) OR (gonadotropin-releasing hormone) OR (buserelin) OR (goserelin) OR (leuprolide) OR (nafarelin) OR (triptorelin)). In addition, the manual-searched method was performed to recruit more studies that had the potential to be eligible among references of articles or similar reviews.

Two reviewers (P.C. and N.J.) independently screened the titles and abstracts of each trial to retrieve interesting articles. Subsequently, full-text articles were contemplated being possibly eligible, and then these trials were scrutinized for eligibility. The references listed in previous meta-analyses were checked and compared with our search, ensuring that all related studies were found. Consensus with the participation of another reviewer (U.S.) was made if there was a disagreement between the reviewers.

### Data Collection and Process

Two reviewers independently completed data extraction from the included trials. When a multiple records study was identified, the most recent and most detailed published data was chosen. We tried to contact the corresponding authors of each trial by e-mail if more information from their trials was required.

The extracted data included authors, institution or center, country, study period, ethical approval, source of funding, conflicts of interest, randomization method, study design, enrollment period, eligibility criteria, exclusion criteria, the number of participants, mean age, body mass index, the number of embryos transferred per woman, endometrial preparation method and regimen of luteal phase support. Clinical pregnancy is our primary outcome which is characterized by the detection of an intrauterine gestational sac with or without ultrasound-confirmed fetal heart activity. Secondary outcomes included chemical pregnancy or other terms that suggest similar meanings such as positive pregnancy and positive beta-human chorionic gonadotropin (β-hCG), ongoing pregnancy, miscarriage or abortion, live birth, and extrauterine pregnancy.

### Risk of Bias in Individual Studies

Two reviewers (P.C. and N.J.) assessed the risk of bias independently by following a revised Cochrane risk of bias tool for randomized trials (RoB 2) (Sterne *et al.,* 2019). RoB 2 assessment of five domains of bias included the following 1) Risk of bias arising from the randomization process, 2) Risk of bias due to deviations from the intended interventions, 3) Risk of bias due to missing outcome data, 4) Risk of bias in the measurement of the outcome and 5) Risk of bias in the selection of the reported result. Overall risk-of-bias judgments among studies were classified as “low risk of bias”, “some concerns”, or “high risk of bias”. Disagreements between the two reviewers were reconciled by consensus.

### Statistical Analysis

All data were pooled and analyzed using Review Manager version 5.4.1 software (The Cochrane Collaboration, United Kingdom, 2020). The effects of the interventions from each eligible study were reported as the risk ratio (RR) and the 95% CI was used to evaluate the precision of the estimates. The heterogeneity analysis between studies was assessed by the *I*^2^ test. *I*^2^ less than 50% indicated low heterogeneity.

## RESULTS

### Search Results

**Figure 1:**
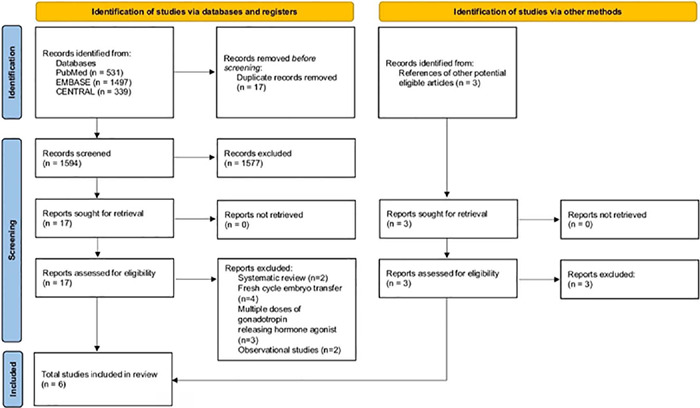
Prisma flow diagram of study identification and selection.

Of the 1594 publications initially identified. Following the removal of duplicated and excluded studies, six eligible RCTs were included for analysis. A PRISMA flow diagram of study identification and selection is depicted in Figure 1.

### Description of the Studies

The characteristics of the six RCTs are illustrated in Table 1. All studies reported clinical pregnancy (Ben-Ami *et al.,* 2015; Davar *et al.,* 2015; Seikkula *et al.,* 2016; 2018; Ye *et al.,* 2019; Wang *et al.,* 2021), and five studies reported chemical pregnancy (other terms; positive pregnancy rate (Seikkula *et al.,* 2016; 2018), beta-hCG positive rate (Ye *et al.* , 2019), and biochemical pregnancy rate (Wang *et al.* , 2021) and abortion (Davar *et al.* , 2015; Seikkula *et al.* , 2016; 2018; Ye *et al.* , 2019; Wang *et al.* , 2021). Four studies reported implantation (Ben-Ami *et al.* , 2015; Davar *et al.* , 2015; Ye *et al.* , 2019; Wang *et al.* , 2021). Two studies and other two studies reported ongoing pregnancy (Davar *et al.* , 2015; Ye *et al.* , 2019), and live birth (Seikkula *et al.* , 2016; 2018), respectively. Miscarriage and extrauterine pregnancy were reported in five (Davar *et al.,* 2015; Seikkula *et al.,* 2016; 2018; Ye *et al.* , 2019; Wang *et al.* , 2021), and three studies (Seikkula *et al.* , 2016; 2018; Wang *et al.* , 2021), respectively.

**Table 1:** Characteristics of Included Randomized Controlled Trials

Study (year)	Patient population	Protocol FET	Luteal phase support + GnRHa	Results
Davar *et al.* (2015)	- Excluded women < 18 and > 40 years of age, oocyte recipients, systemic or endocrine disorders, endometriosis, submucous fibroids, intrauterine adhesions	- Artificial cycle - Embryo stage not reported - Mean number of embryos: 2.38 - 2.45	- Triptorelin 0.1 mg 3 days after transfer - Progesterone 800 mg suppo + estradiol valerate 6 mg + aspirin 80 mg	CPR: 26% *vs.* 21%, p=0.40 Ong. PR: 22% vs. 17%, p=0.37 Imp. Rate: 14.66% vs. 13.07%, p=0.31 Abor. Rate: 5% *vs.* 8%, *p*=0.39 Chem. PR: 27% *vs.* 27%, *p*=1.00
Seikkula *et al.* (2016)	- Excluded women age > 42 years, chromosomal abnormality, testicular or donated sperm used without female cause of infertility, donated oocytes used, congenital uterine anomalies, intramural myomas > 4 cm, submucous myoma, endometrial polyp > 1 cm, endometrium thickness < 6 mm before embryo transfer, blastocyst transfer, untreated thyroid dysfunction or hyperprolactinemia, allergy to triptorelin	- Natural cycle - Cleavage-stage embryos - Mean number of embryos: 1.1	- Triptorelin 0.1 mg at age 6 days of the transferred embryos - Progesterone 400 mg/day	LBR: 20.8% vs. 24.2%, *p*=0.481 CPR: 38.5% *vs.* 27.4%, *p*=0.199 PPR: 43.1% vs. 32.3%, *p*=0.212 Misc. rate: 12% *v*s. 1.8%, *p*=0.892 Extrauterine PR: 8% *vs.* 0%. *p* value N/A
Seikkula *et al.* (2018)	- Excluded women age > 42 years, chromosomal abnormality, testicular or donated sperm used without female cause of infertility, donated oocytes used, congenital uterine anomalies, intramural myomas > 4 cm, submucous myoma, endometrial polyp > 1 cm, endometrium thickness < 6 mm before embryo transfer, untreated thyroid dysfunction or hyperprolactinemia, allergy to triptorelin	- Artificial cycle - Blastocyst embryos - Mean number of embryos: 1.1	- Triptorelin 0.1 mg at age 6 days of the transferred embryos - Micronized progesterone 600 mg vaginally	LBR: 29.2% *vs.* 19.4%, *p*=0.11 CPR: 40.3% *vs.* 36,1%, *p*=0.693 PPR: 45.8% *vs* 41.7%, *p* = 0.691 Misc. rate: 27.6% *vs.* 42.3%, *p*=0.535 Extrauterine PR: 0% vs. 3.8%, *p* value N/A
Ye *et al.* (2019)	- Included women aged 20-37 years, BMI 28 kg/m2, 1 good quality embryo of day 3 after thawing - Excluded oocyte recipients, uterine abnormalities, intrauterine adhesions with or without a history of previous surgery, endometrial thickness ≤ 7 mm on starting day of progesterone administration	- Artificial cycle - A day 3 after thawing embryos - Mean number of embryos: 2.0 ± 0.1	- Triptorelin 0.1 mg 3 days after transfer - Progesterone 100 mg injection/day	Imp. rate: 41% *vs.* 39.2%, *p*=0.476 CPR: 57.7% *vs.* 58.7%, *p*=0.781 Ong. PR: 46.7% vs. 49.3%, *p*=0.478 Misc. rate: 11% vs. 9.9%, *p*=0.673 β-hCG rate: 68.8% vs. 68.4%, *p*=0.914
Wang *et al.* (2021)	- Included women aged 20-45 years old, who have previous IVF/ICSI cycle, had at least 1-2 blastocyte-stage embryo(s) cryopreserved freezing, had undergone FET cycles - Excluded women with infertility > 10 years, chromosomal abnormalities, hydrosalpinx, uterine malformations, submucosal myoma, history of tuberculosis or any uncontrolled endocrine disorder that may affect pregnancy, history of endometrial hyperplasia	- Natural and artificial cycles - Blastocyst embryos - Number of embryos: 1-2	- Triptorelin 0.1 mg on the day of transfer - Other luteal support not clarified	CPR: 56.3% *vs.* 50.58%, *p*=0.086 Imp. rate: 39.98% *vs.* 38.01%. *p*=0.425 Biochem. PR: 15.87% vs. 18.94%, *p*=0.23 Ectopic PR: 0.43% *v*s. 0.69%, *p*=0.947 Abor. Rate: 10.43% *vs.* 12.01%, *p*=0.46
Ben-Ami *et al.* (2015)	Patients undergoing a cleavage stage FET	- Natural cycle - Cleavage stage - Number of embryos: ≥ 2	- Triptorelin 0.1 mg 3 days after transfer - hCG 2,500 IU every 3 days x 4 injections	PR: 57.4% vs. 31.8%, *p*=0.02 CPR: 51% vs. 27.2%, *p*=0.029 Imp. rate: 25.2% *vs.* 13.6%, *p*=0.036

FET, frozen-thawed embryo transfer; GnRHa, gonadotropin releasing hormone agonist; BMI, body mass index; IVF, *in vitro* fertilization; ICSI, intracytoplasmic insemination; hCG, human chorionic gonadotropin; CPR, clinical pregnancy rate; Ong. PR, ongoing pregnancy rate; Imp., implantation; Abor., abortion; Chem. PR, chemical pregnancy rate; Biochem. PR, biochemical pregnancy rate; LBR, live birth rate; PPR, positive pregnancy rate; Misc., miscarriage; PR, pregnancy rate; N/A, not available.

However, one study (Ben-Ami *et al.* , 2015) could be found only a published abstract online. Unfortunately, we were unsuccessful in having their full paper, resulting in the lack of in-depth details.

According to the six studies, the implantation rate was reported in four studies. Davar *et al.* (2015) reported the implantation rate in terms of several cycle transfers, whereas others provided a unit of these parameters as the total number of participants. With our efforts, we could not receive more data on the cycle and patient numbers for extraction since no raw data were available. Consequently, only three studies were included to evaluate the effect on implantation rate (Ben-Ami *et al.* , 2015; Ye *et al.* , 2019; Wang *et al.*, 2021).

### Risk of Bias

**Figure 2:**
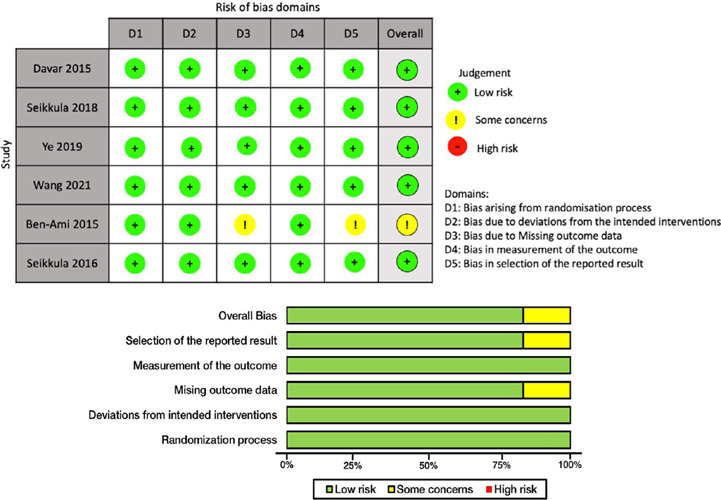
Results of risk of bias assessment using RoB 2.

In overall bias, five of six RCTs were rated as “low risk” (83.3%), whereas the other reported “some concerns” for overall bias and reporting bias and attrition bias (16.7%). All RCTs had a “low risk of bias” for randomization, allocation, and outcome measurement (Figure 2).

### Outcomes

#### Clinical Pregnancy Rate

**Figure 3:**
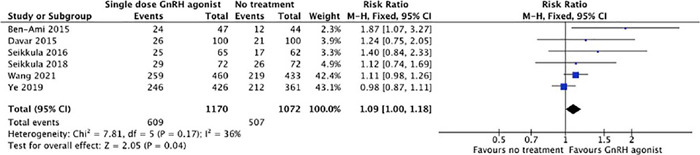
Forest plot for meta-analysis on clinical pregnancy rate from six studies.

A significantly higher rate of pooled clinical pregnancy was observed in the GnRHa group than the control group (52.05% [609/1170] *vs.* 47.29% [507/1072]; p=0.04; RR=1.09; 95% CI = 1.00 -1.18; *I*^2^ = 36%; Figure 3).

**Figure 4:**
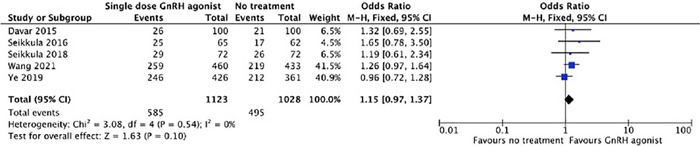
Forest plot for meta-analysis on clinical pregnancy rate from five studies, excluding Ben-Ami *et al.* (2015).

Because of some concerns about overall bias, recalculated pooled clinical pregnancy rate from five studies (Davar *et al.,* 2015; Seikkula *et al.,* 2016; Seikkula *et al.,* 2018; Ye *et al.,* 2019; Wang *et al.,* 2021) was done, and it could not show statistical differences between the groups (52% [585/1123] *vs.* 48.1% [495/1028]; p=0.10; RR=1.15; 95% CI=0.97-1.37; Figure 4).

**Figure 5:**
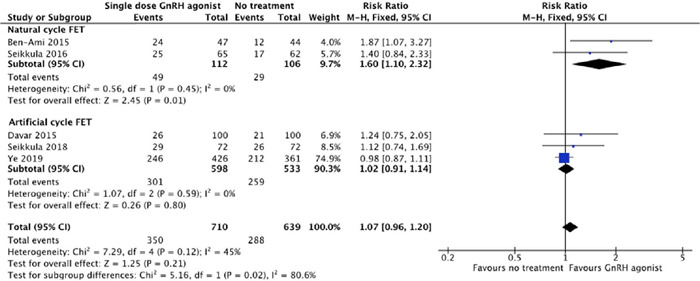
Forest plot for meta-analysis on clinical pregnancy rate. Subgroup analysis according to endometrial preparation protocol for FET between artificial and natural cycles.

Five studies were included in subgroup analyses to determine the endometrial preparation protocol. A subgroup analysis for the natural cycle (Ben-Ami *et al.,* 2015; Seikkula *et al.,* 2016), the clinical pregnancy rate in the interventional group was significantly higher compared with that in the control group. (43.75% [49/112] *vs.* 27.35% [29/106]; p=0.01; RR=1.6; 95% CI = 1.10-2.32; Figure 5). Conversely, for the artificial cycle (Davar *et al.,* 2015; Seikkula *et al.,* 2018; Ye *et al.,* 2019), the clinical pregnancy in the GnRHa group 50.33% (301/598) did not show a significant difference to the clinical pregnancy in the control group 48.59% (259/533%). The statistical outcome was a RR value of 1.07 (95% CI=0.96-1.20; Figure 5).

**Figure 6:**
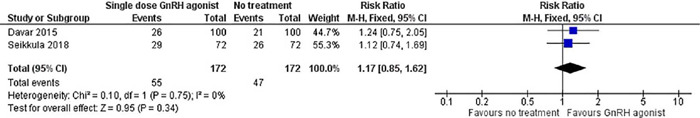
Forest plot for meta-analysis on clinical pregnancy rate from the studies using vaginal progesterone for luteal phase support.

A subgroup analysis for the vaginal route progesterone (Davar *et al.,* 2015; Seikkula *et al.,* 2018), no significant difference in clinical pregnancy rate was observed between the groups. (p=0.34; RR=1.17; 95% CI=0.85-1.62; Figure 6).

#### Chemical Pregnancy Rate

**Figure 7:**
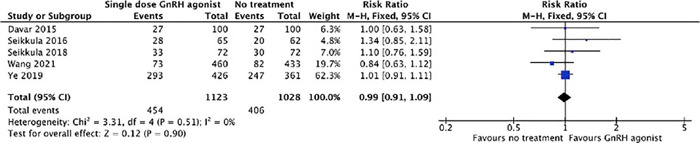
Forest plot for meta-analysis on chemical pregnancy rate.

The pooled chemical pregnancy rate was comparable between the groups (40.4% [454/1123] *vs.* 39.5% [406/1028]; p=0.9; RR=0.99; 95% CI = 0.91-1.09; I^2^=0%; Figure 7).

#### Ongoing Pregnancy Rate

**Figure 8:**
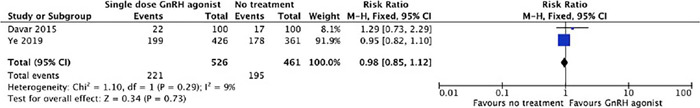
Forest plot for meta-analysis on ongoing pregnancy rate.

The pooled ongoing pregnancy rate was comparable between the groups (42.0% [221/526] vs. 42.3% [195/461]; p=0.73; RR=0.98; 95% CI = 0.85-1.12; *I*^2^=9%; Figure 8).

#### Implantation Rate

**Figure 9:**
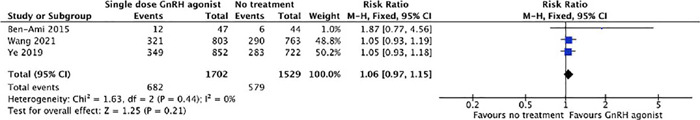
Forest plot for meta-analysis on implantation rate.

Due to unavailable raw data, only three studies were included to evaluate the effect on implantation rate. The pooled implantation rate was comparable between the groups (40.0% [682/1702] *vs.* 37.8% [579/1529]; *p*=0.21; RR=1.06; 95% CI=0.97-1.15; *I*^2^=0%; Figure 9).

#### Live Birth Rate

**Figure 10:**
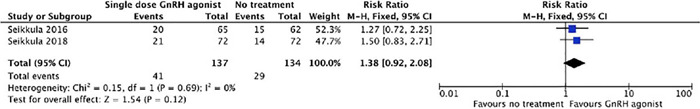
Forest plot for meta-analysis on live birth rate.

The pooled live birth rate showed higher live birth rates in the GnRHa group compared with that in the group without GnRHa (29.9% [41/137] vs. 21.6% [29/134]; *p*=0.12; RR=1.38; 95% CI=0.92-2.08; *I*^2^=0%; Figure 10).

#### Miscarriage Rate

**Figure 11:**
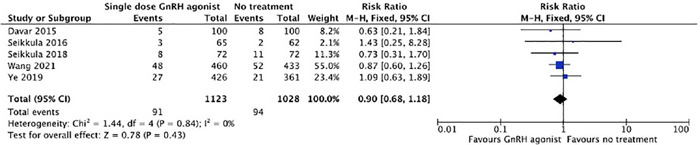
Forest plot for meta-analysis on miscarriage rate.

The pooled miscarriage rate did not differ between the groups (8.1% [91/1123] vs. 9.1% [94/1028]; *p*=0.43; RR=0.90; 95% CI=0.68-1.18; *I*^2^=0%; Figure 11).

#### Extrauterine Pregnancy Rate

**Figure 12:**
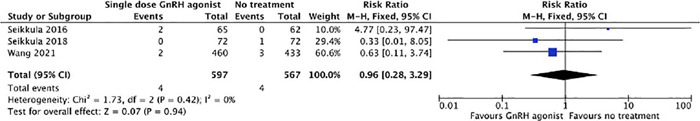
Forest plot for meta-analysis on extrauterine pregnancy rate.

The pooled extrauterine pregnancy rate was not different between the groups (0.6% [4/597] vs. 0.7% [4/567]; *p*=0.94; RR=0.96; 95% CI = 0.28 −3.29; *I*^2^=0%; Figure 12).

## DISCUSSION

The exact mechanisms of the positive effect of GnRHa during the luteal phase on pregnancy outcomes remain to be elucidated. GnRHa may directly affect either endometrial stroma and cells or preimplantation embryos. It has been shown that GnRH gene expression and messenger ribonucleic acid (mRNA) of GnRH receptors appear in the human placenta and regulate hCG production throughout pregnancy (Lin *et al.,* 1995). Besides, GnRH also plays a role in regulating the balance between tissue-specific inhibitors of matrix metalloproteinases and matrix metalloproteinases expression in decidual cells (Chou *et al.,* 2003).

Certainly, GnRHa was described in several studies regarding the improvement of endometrium receptivity and embryo development (Raga *et al.,* 1998; Casañ *et al.*, 1999; Nam *et al.*, 2005; Li *et al.*, 2022).There are several GnRHa studies, which show pregnancy outcomes. A study showed a higher implantation rate after a single-dose GnRHa injection, six days following ICSI of donated oocytes but no difference in pregnancy rate (Tesarik *et al.,* 2004). In 2006, another study from previous investigators conducted subsequent trials with a similar protocol with autologous oocytes and then reported a significantly greater implantation and birth rate in the GnRHa group (Tesarik *et al.*, 2006). Ata et al. reported no improvement in any pregnancy outcomes after adding a single dose of triptorelin 0.1 mg six days after ICSI following the long GnRHa protocol of ovarian stimulation (Ata *et al.*, 2008).

Our present study evaluated six RCTs including a total of 2242 participants. The meta-analysis results reveal a marginal benefit on clinical pregnancy rate in participants receiving GnRHa. Nevertheless, regardless of one RCT with limited data, the clinical pregnancy rate was comparable between patients in the GnRH group and those in the control group. Lately, there has been a randomized clinical pilot study that recruited 156 patients to investigate the effect of the extra single dose of GnRHa (Liu *et al.,* 2023). The authors concluded that insignificant differences in all pregnancy outcomes of artificial cycle frozen embryo transfers were observed. We estimate that our pregnancy outcomes would not improve considerably if data from the recent RCT is involved.

Our results displayed a significantly higher clinical pregnancy rate among the GnRH group with the natural cycles. In the case of existing corpus luteum development in the non-artificial endometrial preparation, the additional GnRHa injection may aid progesterone secretion during early pregnancy. GnRHa supplement plausibly serves to restore luteal phase function, which could affect serum LH levels, thereby maintaining corpora lutea (Fujii *et al.,* 2001; Pirard *et al.* , 2005).

Contrary to previous evidence, GnRH acts as a luteolytic factor by promoting apoptosis in luteinized granulosa cells, resulting in decreased progesterone release (Metallinou *et al.*, 2007). Moreover, studies suggested that desensitization of GnRH receptors could occur by a luteolytic effect of GnRHa, and the use of GnRHa led to a decline in the functioning of the corpus luteum (Lemay *et al.,* 1983; Herman *et al.,* 1992).

In the artificial cycle, hormonal replacement for endometrial preparation may impair endometrial receptivity by earlier closure of the implantation window at high estradiol levels (Wu *et al.,* 2021). In vaginal progesterone used, the subgroup analysis demonstrated no beneficial effect of additional GnRHa administration. Since only two trials with a small number of participants were included, it is still unclear whether the route of progesterone affects pregnancy outcomes.

The definition in this study followed the International Glossary on Infertility and Fertility Care in 2017 was used for diagnosing the clinical pregnancy by ultrasonographic visualization of one or more gestational sacs or definitive clinical signs of pregnancy regardless of fetal heart activity (Zegers-Hochschild *et al.,* 2017). This would cover all clinical pregnancy terminology in our six RCTs, which had different terminology of clinical pregnancy. Consequently, it may have an impact on clinical pregnancy rates by incorporating abnormal early pregnancies such as blighted ova.

For secondary outcomes, the intervention group had greater normal pregnancy rates than the control group insignificantly. Similarly, the rates of unfavorable pregnancies, including extrauterine pregnancy and loss, did not differ between groups.

There are two strengths in our study. First, we intend to pool only RCT-designed studies, which are statistically considered to be the highest quality of evidence among all types of clinical studies. Additionally, our meta-analysis can demonstrate a “low risk” of overall bias among the studies. Second, despite the small number of studies for analysis, no significant heterogeneity was observed across the studies. This indicated that the majority of studies had no variation in their findings.

This study has certain limitations. The number of eligible studies and the total number of participants were both small. Inevitably, we chose clinical pregnancy as our primary endpoint rather than live birth, which is widely regarded as the best performance indicator for ART methods.Differences in progesterone regimens for improving the luteal phase receptive endometrium could theoretically alter pregnancy rates (Vuong *et al.,* 2021; Greenbaum *et al.,* 2022). Transferred embryo quality and number per cycle varied. High-quality or euploid embryos enhance clinical pregnancy rates higher than unqualified embryos (Jimenez *et al.,* 1997; Veleva *et al.,* 2013). These can also have a significant effect on pregnancy outcomes and have been recognized as a weakness of this study.

## CONCLUSION

This RCT-only meta-analysis is significant in that it analyzes the effect of a single-dose GnRHa administration for luteal phase support during the FET cycle. The findings implied that an additional single-dose GnRHa administration benefits clinical pregnancy rates, especially in the natural cycle FET, but did not affect other pregnancy outcomes. According to our research, further high-quality randomized controlled trials are required.
